# Modern Standard Arabic mood changing and depression dataset

**DOI:** 10.1016/j.dib.2022.107999

**Published:** 2022-03-01

**Authors:** Ashwag Maghraby, Hosnia Ali

**Affiliations:** Computer Science Department, College Of Computer Science and Information, Umm Al-Qura University, Mecca, Saudi Arabia

## Abstract

This paper presents Modern Standard Arabic data for the automatic estimation of the risk of depression for online personas based on their daily Arabic tweets. The data were collected from 1-1-2020 to 1-1-2021 using automatically collected samples of depression and non-depression tweets. The data contain 1229 records. These data can be used to develop machine-learning tools to identify the risk of an individual being depressed and to build recommender systems that monitor depression.

## Specifications Table

**Table:** 

Subject	Data science
Specific-subject area	Data science
Type of data	.csv file
How data were acquired	Using TWINT tool from python
Data format	Raw data (.csv)
Description of data collection	The raw data of Twitter posts were downloaded using the TWINT tool from Python. The collection process was conducted from 1-1-2020 to 1-1-2021. An Arabic version of the Patient Health Questionnaire (PHQ9) scale was used to specify the categories of the depression dataset. The categories are: (1) losing interest or pleasure in activities, (2) low mood, (3) sleep disorder, (4) loss of energy, (5) weight disorder, (6) feelings of worthlessness, (7) diminished ability to think or concentrate, (8) psychomotor agitation or retardation, and (9) suicidality. Keywords (terms or phrases) that might fall under each of the categories were created, including terms or phrases a person would use to describe their moods, anxiety, and sleep modules, such as “حزينة”,“ فيني أرق ” and “يا رب حياتي تنتهي” These keywords were then used to collect relevant tweets from Twitter.
Data source location	Middle East
Data accessibility	Repository name: Mendeley data Data identification number: 10.17632/myrb2gky8w.1 Direct URL to the dataset: https://data.mendeley.com/datasets/myrb2gky8w/draft?a=660d1700-1694-4475-8a9a-ed50f538b99d

## Value of the Data

• This dataset contains information about real users of Arabic tweets to acquire knowledge about a person's psychology. It can be used for the early detection of depression types for rapid intervention, which can potentially reduce the suffering from the disorder and the stigma associated with mental illness.
• This dataset provides a unique opportunity for researchers, primary care clinics, and psychologists to detect users’ depression early (12 different mental health disorders, including depressed mood, loss of interest, change in appetite or weight, feeling guilty or worthless, sleep disorder, psychomotor agitation or impairment, fatigue or loss of energy, poor concentration and persistent thoughts of death or suicidal ideation) based on their last 14 days of tweets.
• This dataset can be used to automatically complete the Patient Health Questionnaire (PHQ-9), the most well-known psychometric symptoms report for measuring depression symptoms and mental illnesses [1].
• This dataset can help researchers easily build machine-learning and classification models to detect users’ depression types.
• This is a language-dependent dataset designed for the Arabic language with language-dependent features. However, researchers can recreate a similar dataset for other languages, such as English, by following the same steps explained in this work.

## 1 Data Description

**Fig. 1:**
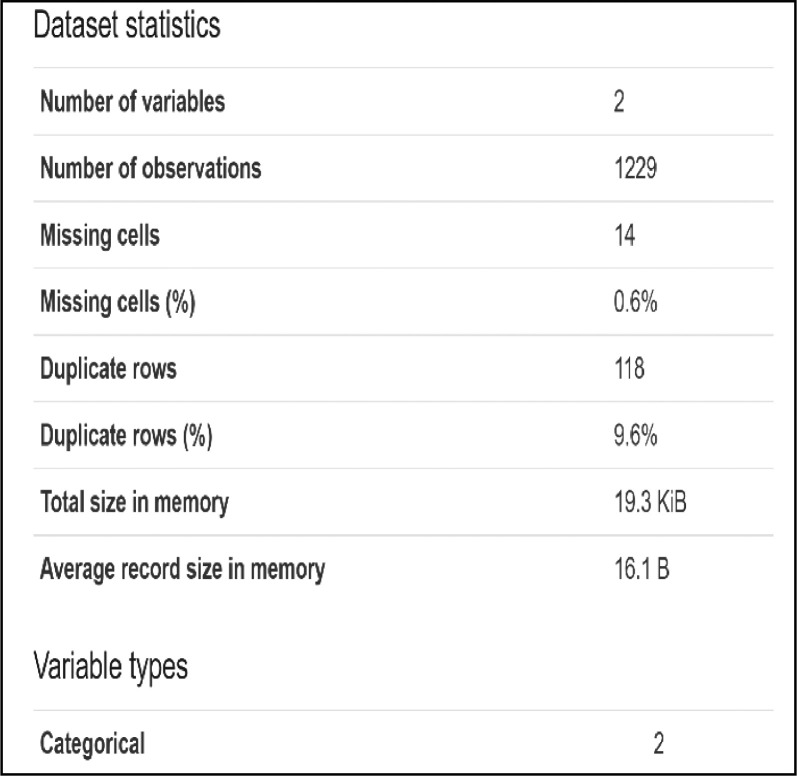
Overview of the final dataset.

**Fig. 2:**
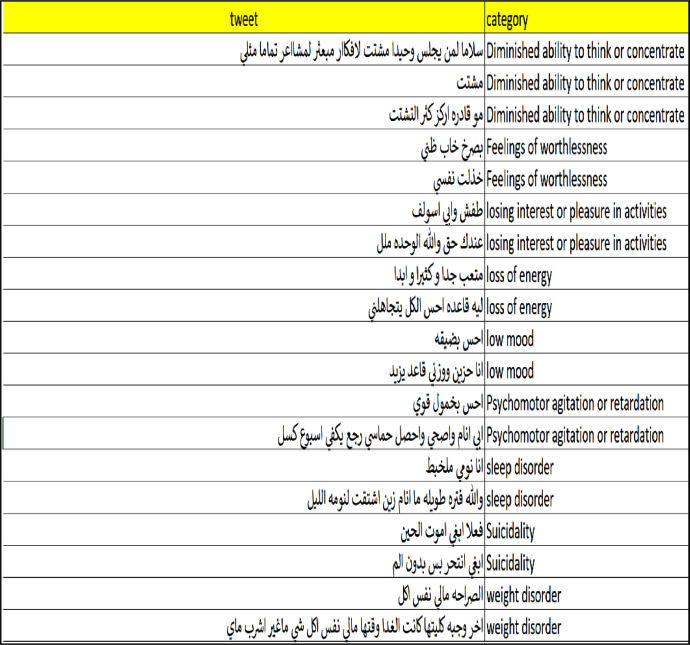
Sample of the final dataset.

**Fig. 3:**
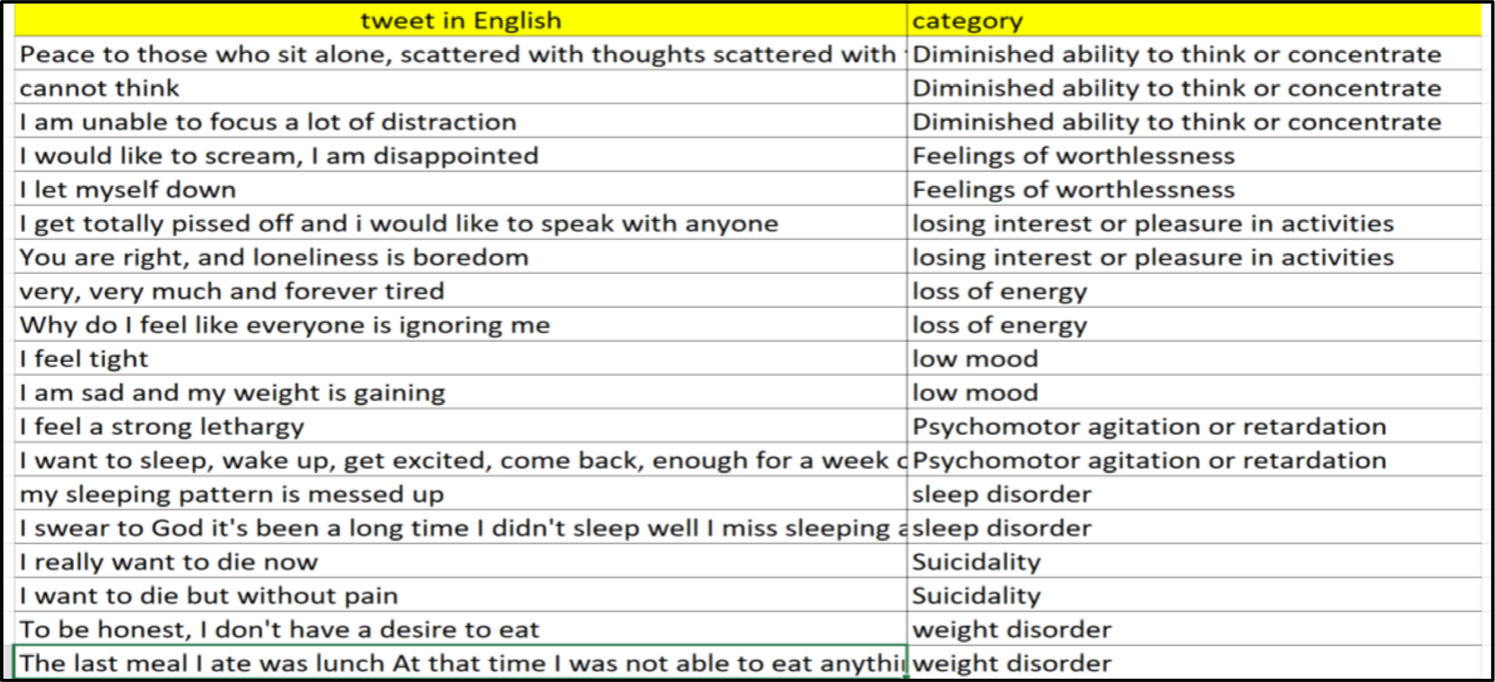
Sample of the final dataset in English.

This paper presents Modern Standard Arabic data for the automatic estimation of the risk of depression for online personas based on their daily Arabic tweets. The data were collected from 1-1-2020 to 1-1-2021 using automatically collected samples of depression and non-depression tweets. The final data contain 1229 records. These data can be used to develop machine-learning tools to identify the risk of an individual being depressed and to build recommender systems that monitor depression Fig. 1 shows the final dataset overview, and Fig. 2 shows a sample from the final dataset Fig. 3 shows a English version of Fig. 2.

## 2 Experimental Design and Materials and Methods

An Arabic version of the Patient Health Questionnaire (PHQ-9) scale [1], a well-validated measurement tool for depression, was used in this manuscript. Although it cannot be used formally as a standalone assessment to diagnose clinical depression, it can be used as the first step to screen and detect depression [2], [3]. It is commonly used in depressive symptoms self-administered identification tools, online design studies, research, and psychophysical clinics during the first appointment of subjects.

Subjects are required by PHQ-9 to respond by one of the following numbers: 0, 1, 2, or 3 to nine psychophysical questions that indicate depression. Each of these numbers indicates a certain degree of depression, as shown in Table 2. The PHQ9 scale questions are shown in Table 1. In these questions, users are asked about their psychophysical state in the last two weeks (14 days), emotions (e.g., feeling down, tired, tired, anxious), appetite, the occurrence of any sleeping disorder, slowness in movement, and suicidal thoughts [1].

**Table 1:** Patient health questionnaire-9 (PHQ-9).

No.	English/Arabic Questions	0	1	2	3
1	Little interest or pleasure in doing things? قلة الاهتمام أو قلة الاستمتاع بممارسة بالقيام بأي عمل				
2	Feeling down, depressed, or hopeless? الشعور بالحزن أو ضيق الصدر أو اليأس				
3	Trouble falling or staying asleep, or sleeping too much? صعوبة في النوم أو نوم متقطع أو النوم أكثر من المعتاد				
4	Feeling tired or having little energy? الشعور بالتعب أو بامتلاك القليل جداً من الطاقة				
5	Poor appetite or overeating? قلة الشهية أو الزيادة في تناول الطعام عن المعتاد				
6	Feeling bad about yourself — or that you are a failure or have let yourself or your family down? الشعور بعد الرضا عن النفس أو الشعور بأنك قد أخذلت نفسك أو عائلتك				
7	Trouble concentrating on things, such as reading the newspaper or watching television? صعوبة في التركيز مثلاً أثناء قراءة الصحيفة أو مشاهدة التلفزيون				
8	Moving or speaking so slowly that other people could have noticed? Or so fidgety or restless that you have been moving a lot more than usual? بطء في الحركة أو بطء في التحدث عما هو معتاد لدرجة ملحوظة من الآخرين أو العكس من ذلك التحدث بسرعة وكثرة الحركة أكثر من المعتاد				
9	Thoughts that you would be better off dead, or thoughts of hurting yourself in some way? راودتك أفكار بأنه من الأفضل لو كنت ميتاً أو أفكار بأن تقوم بإيذاء النفس.

**Table 2:** Valid values for PHQ-9.

Value	Value Meaning	Description	Display Order
0	Not at all	A subjective answer of non-agreement.	0
1	Several days	An indefinite small quantity that is more than two but less than many.	1
2	More than 1/2 the days	A time period that includes one-half of the total number of days plus at least one more.	2
3	Nearly every day	Not quite or just short of: Including all possibilities or occurring at regular intervals.	3

PHQ-9 scores have been shown in previous studies to be strongly correlated with the results of psychologist depression diagnosis in the psychophysical clinic [4]. Generally, subjects with scores ranging from 0 to 9 are considered healthy, subjects with scores ranging from 10 to 19 are considered to have mild depression, and subjects with scores of 20 points or above are considered severely depressed.

Although depression detection is the first step in treating it [2], more than a quarter of most depression patients remain undiagnosed [3]. The main issue is that most patients dislike traditional depression detection methods, such as the paper version of the PHQ-9 scale. In summary, subjects find the questionnaire intrusive, cumbersome, and feared [5].

Twitter data were collected in this work to deal with this issue. It used to automatically answer the nine questions of PHQ-9 for depression screening and detection. Researchers can use this dataset to develop an automatic mental health assessment methodology in which the depression levels of the subjects are detected.

This work began by creating categories for each of these questions, as shown in Table 3. We then chose keywords (terms or phrases) that could fall under each of the categories. The keywords included terms or phrases a person would use to describe their moods, anxiety, and sleep modules, as shown in Table 4. The individual to be diagnosed with depression must experience five or more symptoms during the last two-week (14 days) period of the following nine categories: losing interest or pleasure in activities, low mood, sleep disorder, weight disorder, loss of energy, feelings of worthlessness, diminished ability to think or concentrate, psychomotor agitation or retardation, and suicidality [1]. These keywords were used to collect relevant tweets from Twitter using the TWINT library as follows [6]:

• Python programming language with the TWINT library was used to collect relevant tweets.
• Up to 10000 tweets were scraped for each category.
• Tweets were generated by 38101 Saudi Twitter users
• Data were scraped from 1-1-2020 to 1-1-2021.
• Records with multiple attributes (id, conversation_id, created_at, date, time, timezone, user_id, username, name, place, tweet, language, mentions, urls, photos, replies_count, retweets_count, likes_count, hashtags, cashtags, link, retweet, quote_url, video, thumbnail, near, geo, source, user_rt_id, user_rt, retweet_id, reply_to, retweet_date, translate, trans_src, trans_dest) were scraped.

**Table 3:** PHQ-9 categories.

No.	English PHQ9 Categories	Arabic PHQ9 Categories
1	Losing interest or pleasure in activities	قلة الاهتمام أو قلة الاستمتاع بممارسة بالقيام بأي عمل Little interest or pleasure in doing things?
2	Low mood	الشعور بالحزن أو ضيق الصدر أو اليأس Feeling down, depressed, or hopeless?
3	Sleep disorder	صعوبة في النوم أو نوم متقطع أو النوم أكثر من المعتاد Trouble falling or staying asleep, or sleeping too much?
4	Loss of energy	الشعور بالتعب أو بامتلاك القليل جداً من الطاقة Feeling tired or having little energy?
5	Weight disorder	قلة الشهية أو الزيادة في تناول الطعام عن المعتاد Poor appetite or overeating?
6	Feelings of worthlessness	الشعور بعد الرضا عن النفس أو الشعور بأنك قد أخذلت نفسك أو عائلتك Feeling bad about yourself — or that you are a failure or have let yourself or your family down?
7	Diminished ability to think or concentrate	صعوبة في التركيز مثلاً أثناء قراءة الصحيفة أو مشاهدة التلفزيون Trouble concentrating on things, such as reading the newspaper or watching television?
8	Psychomotor agitation or retardation	بطء في الحركة أو بطء في التحدث عما هو معتاد لدرجة ملحوظة من الآخرين أو العكس من ذلك التحدث بسرعة وكثرة الحركة أكثر من المعتاد Moving or speaking so slowly that other people could have noticed? Or so fidgety or restless that you have been moving a lot more than usual?
9	Suicidality	راودتك أفكار بأنه من الأفضل لو كنت ميتاً أو أفكار بأن تقوم بإيذاء النفس. Thoughts that you would be better off dead, or thoughts of hurting yourself in some way?

**Table 4:** Keywords for the PHQ-9 categories.

Categories No.	Keywords
1	متبلد - متبلده – طفش – زهق - مو مهتم - مو مهتمة - الأيام متشابهة – مو فارق معي – مو فارقه معي – الحياة مالها طعم – مو فارق معاي – مو فارقة معاي – مو مستمتع – مو مستمتعة – ملل – أشعر بالملل – الحياة مملة Dull - dull - bored - not interested - the days are the same - no difference with me -life has no taste - not amused - not enjoying - bored - I feel bored - life is boring
2	حزينة – حزين – أنا حزين – أنا حزينة - أشعر بالحزن – غير سعيد – غير سعيدة – ضايق صدري – ضيقة – أشعر بالضيق – أشعر بالضيقة - يأس – يائسة – أنا يئست – أشعر باليأس – حياتي بائسه – أنا تعيس – أنا تعيسة – أشعر بتعاسة – متشائم – متشائمة – شايل هموم – شايلة هموم – منزعج – منزعجه – ضايق علي – ضايقه علي – ضايق فيني – ضايقه فيني – أحس بضيق – أحس بضيقة sad – sad – I am sad – I am sad – I feel sad – unhappy – my chest upset – tight – I feel upset – I feel bad – despair – I despair – I feel despair – my life is miserable – I am miserable – I feel miserable - pessimistic - worrying – annoyed - bothered - annoyed me - I feel distressed
3	ما انام – ما بنام – نومي متقطع – نومي غير منتظم – ما فيني نوم – ما فيا نوم – نومي ملخبط – عندي أرق – فيني أرق – مو قادر أنام – مو قادرة أنام I do not sleep - my sleep is intermittent - my sleep is irregular - I ca not sleep - my sleep is messy - I have insomnia
4	متعب – متعبة – اشعر بالتعب – مجهد – ما في طاقة – ما عندي طاقة – ما فيني طاقة - مو قادر أعمل شيء – ارهاق – اشعر بإرهاق – منهك – منهكة Tired - I feel tired – I am tired no energy - I have no energy - I cannot do anything - tire - I am tired - exhausted
5	مو مشتهي أكل – مو مشتهية أكل – ما اشتهي أكل – مالي نفس أكل – مو قادر أكل – مو قادرة أكل – نفسي مسدودة عن الأكل – ما ابى اكل – ما ابغى أكل I do not crave to eat - I do not want to eat – I cannot eat. I am unable to eat- I am not able to eat -I am blocked from eating
6	مو راضي عن نفسي – مو راضية عن نفسي – خذلت نفسي – خذلت عائلتي – أشعر بخذلان – أشعر بخذل – أحس بخذلان – أحس بخذل – ماني راضي عن نفسي - ماني راضية عن نفسي – أحس بعدم رضا عن نفسي – أحس بعدم رضا – خيبت ظني في نفسي – خيبت ظن أهلي – خيبت ظن عائلتي – خيبت ظن الكثير – خيبت ظن الناس – خاب ظني I am not satisfied with myself - I have let myself down - I have let my family down - I feel let down I am not satisfied with myself - I feel dissatisfied with myself - I feel dissatisfied - I am disappointed In myself – I disappointed my family – I disappointed a lot – I disappointed people - I disappointed myself
7	مو قادرة أركز - مو قادر أركز – تفكيري مشتت – مشتته – مشتت I cannot focus - my thinking is scattered - My attention is distracted
8	أشعر بخمول – أحس بخمول – أعاني من خمول – أحس بكسل – أشعر بكسل – أعاني من الكسل – ثقل اللسان – قلة حركة - كسل – خمول I feel lethargic - I feel lazy - I am lazy - I have laziness - I have a sluggish tongue
9	ابغى اموت – ابى اموت - ابغى انتحر – ابى انتحر - ما ابى الحياة – ما ابغى الحياة - ما ابغى أعيش - ما ابى أعيش - ابغى انهي حياتي – ابى انهي حياتي - ارغب في انتحار – ارغب بالانتحار - اريد الموت - نفسي اموت - يا رب اموت – يا رب حياتي تنتهي - يا رب افارق الحياة - ليتني مت - ليتني ميته - ليتني كنت ميته I want to die - I want to commit suicide - I do not want to live - I want to end my life- I want myself to die - Oh Lord I die - Oh Lord my end my life - I wish I had died - I wish I was dead

The final dataset includes 48,821 records Table 5. shows the number of collected recorders for each category.

**Table 5:** No. of tweets under each category.

No.	Categories	No. of records
1	Losing interest or pleasure in activities	10012
2	Low mood	8019
3	Sleep disorder	7347
4	Loss of energy	9705
5	Weight disorder	319
6	Feelings of worthlessness	2068
7	Diminished ability to think or concentrate	3120
8	Psychomotor agitation or retardation	6162
9	Suicidality	2069

**Total records**		48,821 records

### 1.1 Pre-processing dataset

In this stage, three steps were followed: (1) labeling dataset, (2) cleaning dataset, and (3) reviewing dataset.

#### 1.1.1 Labeling the dataset

Each record is labeled by one category name (there are nine categories, as shown in Table 3).

#### 1.1.2 Cleaning the dataset

**Fig. 4:**
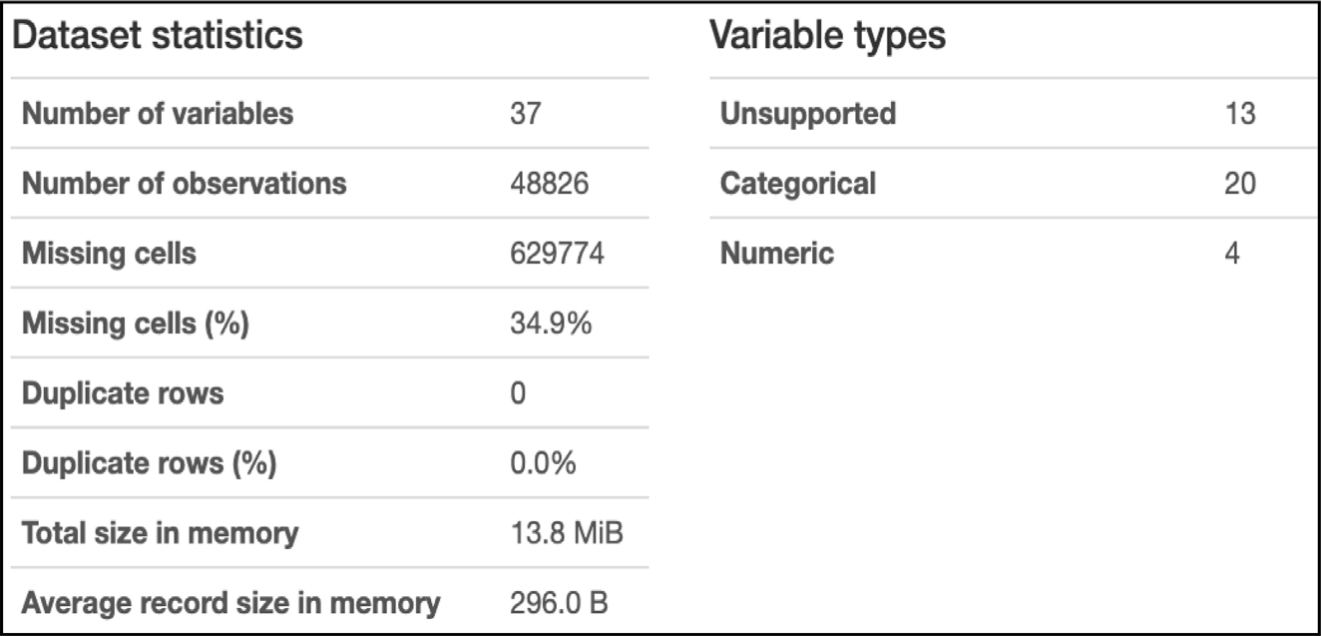
Overview of the dataset before cleaning.

Cleaning is an essential step in almost any Natural Language Processing (NLP) task. It aims to eliminate incomplete, noisy, and inconsistent data. The dataset was analyzed before cleaning using Pandas Profile Report tools Fig. 4. shows the results of this analysis. According to this figure, the biggest problem with this dataset is missing cells (missing values) at 34.9%. Therefore, two cleaning steps were followed.

**Cleaning the dataset file:**

• **Removing empty columns:** removed 10 empty columns that did not contain any value.
• **Removing unwanted columns:** removed 24 unwanted columns that did not contain any important value.
• **Removing duplicated records:** removed duplicated rows (0.04%).

**Fig. 5:**
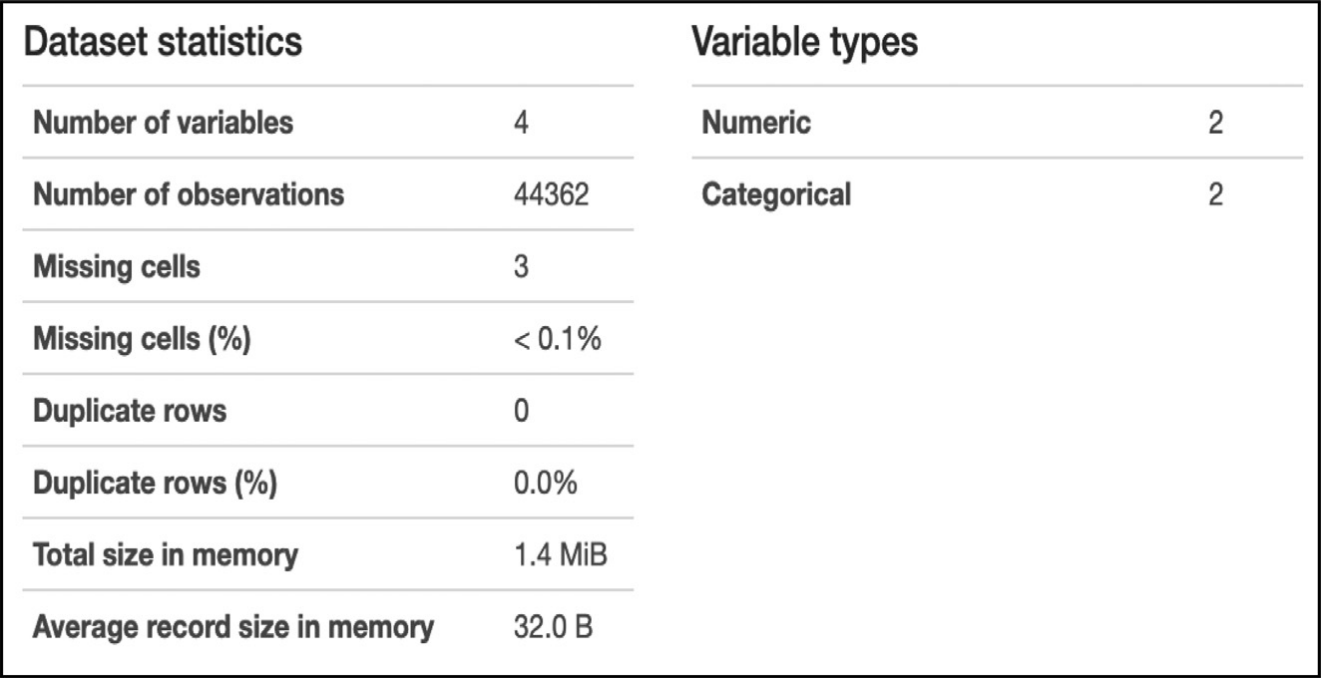
Overview of the dataset after cleaning.

Fig. 5 shows the dataset overview after cleaning the dataset file.

**Cleaning tweets:**

• **Removing URLs:** removed Tweets links that did not contribute to a depression classification.
• **Removing usernames:** Usernames (@user) were also removed from the tweets.
• **Removing duplicated letters:** replaced any letter that appeared consecutively more than two times in a word with one letter.
• **Removing punctuation:** removed punctuation, such as full stop, comma, and brackets.
• **Removing stop words:** removed the most common words in the Arabic language (articles, prepositions, pronouns, conjunctions, etc.) that did not add much information to the text.
• **Removing emojis:** removed all emojis from tweets.
• **Removing English words:** removed all English words.
• **Text normalization:** Transformed a text to a unified form; removed Al-tashkil and elongation.

#### 1.1.3 Reviewing the dataset

Following the cleaning step, each record in the dataset was manually reviewed. Any records that did not relate to depression (e.g., records not describing personal moods, anxiety, movement, sleep mode, suicidal thoughts, or records not associated with the PHQ-9 questions, as shown in Table 1) were disregarded. The number of examples for each category/class label was also balanced to achieve the right accuracy when using machine-learning classification algorithms. The final dataset contains 1229 records of 928 Saudi Twitter users as showing in Fig. 1.

## Ethics Statement

Our data does not provide any personally identifiable information and only the tweet IDs and human annotated stance labels are shared. Thus, all data are fully anonymized and were collected and distributed under Twitter's Developer Policy 2021 [7].

The PHQ-9 is available to healthcare providers completely free of charge and the legal copyright holder, explicitly states that “no permission [is] required to reproduce, translate, display or distribute [the PHQ-9]” [8].

## CRediT authorship contribution statement

**Ashwag Maghraby:** Conceptualization, Methodology, Validation, Investigation, Resources, Data curation, Writing – original draft, Writing – review & editing, Supervision. **Hosnia Ali:** Data curation, Investigation, Resources, Writing – original draft.

## Declaration of Competing Interest

The authors declare that they have no known competing financial interests or personal relationships which have or could be perceived to have influenced the work reported in this article.

## Data Availability

Modern Standard Arabic mood changing and depression dataset (Original data) (Mendeley Data).
